# Activity Profile and Physical Performance of Match Play in Elite Futsal Players

**DOI:** 10.3389/fpsyg.2020.01709

**Published:** 2020-07-24

**Authors:** João Nuno Ribeiro, Bruno Gonçalves, Diogo Coutinho, João Brito, Jaime Sampaio, Bruno Travassos

**Affiliations:** ^1^Department of Sport Sciences, University of Beira Interior, Covilhã, Portugal; ^2^Research Centre in Sport Sciences, Health Sciences and Human Development, CIDESD, CreativeLab Research Community, Vila Real, Portugal; ^3^Departamento de Desporto e Saúde, Escola de Ciências e Tecnologia, Universidade de Évora, Évora, Portugal; ^4^Comprehensive Health Research Centre (CHRC), Universidade de Évora, Évora, Portugal; ^5^Portugal Football School, Portuguese Football Federation, Oeiras, Portugal

**Keywords:** team sports, tracking, physical demands, competition, monitoring

## Abstract

Understanding the physical demands of futsal requires a precise quantification of the players’ activities during match play. This study aimed to (1) describe external load, identifying the differences between the first and second halves in official futsal matches; (2) identify the most important external workload metrics to profile the players; and (3) identify the collinearity between variables in the analysis of physical performance of futsal players. Match external load data were collected from male players (*n* = 28) in six games of the Final Eight of the Portuguese Futsal Cup 2018. The players increased the distance covered per minute at 12–18 km/h in the second half (*p* < 0.01). Dynamic stress load also increased in the second half (*p* = 0.01). The variables that best predicted the physical profile of each player were decelerations (predictor importance, PI = 1), walking (PI = 1), sprinting (PI = 1), jogging (PI = 0.997), total distance covered per minute (PI = 0.992), and metabolic power (PI = 0.989). Decelerations showed the highest association with the clusters levels (*p* < 0.001; PI = 1); this suggests decelerations as a potential candidate for best analyzing the physical load of futsal players. Overall, the data from this exploratory study suggest that distance covered per minute (m/min), number of sprints (>18 km/h), decelerations (greater than-2 m/s), and metabolic power (W/kg) are the variables that most discriminate the load intensity of elite futsal players.

## Introduction

The improvement on technological capability to collect and analyze data has increased the knowledge about load and physical demands of team sports and helps to improve training programs, optimizing performance and reducing the likelihood of injury on top-level players (Fox et al., 2017; Vanrenterghem et al., 2017). Its importance has been recently reinforced, since FIFA has approved the use of specific microsensors and wearable devices in official soccer and futsal matches, opening new perspectives for the understanding of players’ physical performance during competitive scenarios (Roell et al., 2018).

To better perceive the load that players experience during a match, internal load (IL), and external load (EL) should be measured and characterized (Buchheit, 2014; Fox et al., 2017; Clemente et al., 2019). While IL describes the physiological effects of training on the athlete, EL describes the physical demands of training through measures derived from position data, and/or inertial measurement units (IMUs; Gonçalves et al., 2017; Impellizzeri et al., 2019). Actually, the available technology allows establishing individualized performance profiles through the analysis of IL and EL variables and specific algorithms that allow the use of other parameters, like Player Load (PL^TM^), or Metabolic Power (PM^TM^; Bourdon et al., 2017; Polglaze and Hoppe, 2019; Reche-Soto et al., 2019).

External load could be classified into three main categories: (a) kinematics, which quantifies overall movement during exercise; (b) mechanical, which describes a player’s overall load during exercise; and (c) metabolic, which quantifies overall movement energy expenditure during exercise (Rossi et al., 2018). The parameters can also be expressed in absolute (total match time) or relative (effective playing time) terms. Still, with the great amount of data available to measure physical load, the challenge is to understand the most reliable and relevant variables that should be collected to characterize activity profiles of players during training sessions and matches (Buchheit and Simpson, 2017).

The scientific knowledge about EL and activity profiles of futsal players is still scarce (Beato et al., 2017; Naser et al., 2017; Taylor et al., 2017). To the best of our knowledge, only five studies have investigated physical demands data in elite futsal players (official matches): one in the Spanish Professional Futsal League—analysis of distances covered and heart rate (Barbero-Alvarez et al., 2008); one in Australian futsal players—analysis of match demands between levels of competition (Doğramaci et al., 2015); and three in Brazil—analysis of sprints (Caetano et al., 2015), distances covered (De Oliveira Bueno et al., 2014), and distances covered, maximum speeds, and heat maps of player displacements (de Pádua et al., 2017). Additionally, most of these studies were developed specifically for physical testing or during simulated games and only reported average values of some EL parameters. From this perspective, more research is required that accurately inform about the physical load experienced by players (Akenhead and Nassis, 2016), as well as data that may help to quantify it.

In this sense, the present study aimed to characterize the EL of elite futsal match play. In addition, data were computed to identify the external workload metrics that distinguish different futsal players’ profiles. The collinearity between EL variables was also analyzed. We expected to identify different profiles of play according to players’ EL, aiming to improve the understanding of match-play demands in futsal.

## Materials and Methods

### Subjects

Twenty-eight elite male futsal players (age: 24.1 ± 3.4 years) from eight futsal teams that participated in the Final Eight of the Portuguese Futsal Cup 2018 (January 2018) accepted to participate in this study. Inclusion criteria were the following: (1) is a field player; (2) did not report any physical limitations or skeletal muscle injury that could affect performance; and (3) played in both halves in each match. All matches were played in the same neutral indoor multisport court. The study protocol followed the guidelines and was approved by the local Ethics Committee of Universidade da Beira Interior (CE-UBI-Pj-2018-029) and conformed to the recommendations of the Declaration of Helsinki.

### Design

An observational research was used to measure and analyze the EL of players who participated in the Final Eight of the Portuguese Futsal Cup 2018. Four matches in the quarterfinals and two matches in the semifinals of the competition at least 48 h apart were used for the analysis. According to the official futsal rules, two halves of 20 min of effective time were played.

### Methodology

Players’ activity was assessed using IMUs with ultra-wideband (UWB) tracking system technology from WIMU PRO^TM^ (Realtrack Systems, Almeria, Spain). The sampling frequency of WIMUs for the positioning system was 18 Hz. The devices were turned on about 10 to 15 min before the warm-up and placed on players with a specific custom neoprene vest located on the middle line between the scapulae at C7 level. The system has six UWB antennas, placed 4 m outside the court, and operates using triangulation between the antennas and the units to derive the *X* and *Y* coordinates of each unit. Data from the beginning to the end of the match with the exclusion of halftime and time-outs were analyzed using SPRO Software (Realtrack Systems SL, Almeria, Spain). The accuracy and reliability of these devices have been previously reported and validated (Bastida-Castillo et al., 2019).

From positional data, variables were extracted based on the three main categories of EL identified (Rossi et al., 2018): (a) kinematics; (b) mechanical; and (c) metabolic. See Table 1 for details of each variable considered. The absolute and the relative (effective playing time - clock time) values of each variable were calculated.

**TABLE 1:** EL variables recorded in this investigation.

Type	Variable	Sub-variable	Unit	Description
Kinematics	Distance covered (m)	Total	m	Total distance covered in meters
	Relative distance covered (m/min)	Total	m/min	Total distance covered in meters per minute
		Walking	(0–6 km/h) m/min	Total distance covered between 0 and 6 km/h/min
		Jogging	(6.1–12 km/h) m/min	Total distance covered between 6.1 and 12 km/h/min
		Running	(12.1–18 km/h) m/min	Total distance covered between 12.2 and 18 km/h/min
		Sprinting	(18.1–30 km/h) m/min	Total distance covered between 18.1 and 30 km/h/min
	Sprints	Total	SPR/n/min	Frequency > 18 km/h during > 1 s in 1-min window
	Maximum speed (km/h)	Max	Speed_*AVG*_	Average max speed
Mechanical	Impacts (Imp/min)	Total	IMP/n/min	Total impacts recorded per minute above 5 *g* force
	Accelerations	Total	ACC (>2 m/s^2^) n/min	Total positive speed changes per minute
	Decelerations	Total	DEC (> −2 m/s^2^) n/min	Total negative speed changes per minute
	Jumps	Total	JUM/n/min 400-ms flight time	Total number of jumps recorded per minute
	Dynamic stress load (a.u.)	Total	DSL/a.u./min	Total of the weighted impacts of magnitude over 2 *g* per minute
	Player load (a.u)	Total	PL/a.u./min	Accumulated accelerometer load in the three axes of movement
Metabolic	Power metabolic (W/kg)	Total	MP/min	Product of speed and energy cost of the activity derived from inclination and acceleration
	High metabolic load distance (W/kg)	Total	HMLD/min	Distance traveled by a player when the metabolic power is >25.5 W/kg (corresponds to a speed greater than 5.5 m/s or 19.8 km/h)

### Statistical Analysis

Normality of the data was tested with the Kolmogorov–Smirnov test. Since normal distribution was not found in all situations, we used the Wilcoxon rank test to identify differences between each half. Mean ± standard deviation (SD) for full-match data and median (Md) and interquartile range (IR) for the first and second halves were calculated.

A two-step cluster with log-likelihood as the distance measure and Schwartz’s Bayesian criterion was performed to classify athletes according to their performance profiles over the entire match. The analysis was used to classify the players’ performance and to identify the variables that maximized group distances. This method differs from traditional clustering techniques by the handling of categorical variables (assuming variables to be independent), automatic selection of the number of clusters, and scalability (Tabachnick et al., 2007). Through an ANOVA test, variables were ranked according to the predictor’s importance, indicating the relative importance of each predictor in estimating the model (the sum of the values for all predictors on the display is 1). In the functional sense, the predictor importance of each variable provides different weights to support the cluster distribution. A cutoff level of 0.4 was chosen.

Spearman’s correlation test was used to verify the collinearity between variables. Data exploration was conducted based on the correlation matrix that is produced with the “corrplot” function in the R programming language. The criteria adopted to categorize magnitudes of correlations (*r*) were as follows: ≤0.1, trivial; >0.1–0.3, small; >0.3–0.5, moderate; >0.5–0.7, large; >0.7–0.9, very large; and >0.9–1.0, almost perfect (Cohen et al., 2013).

Correlograms were used, with the intensity of the color increasing as the correlation moves further away from zero. Here, the correlation coefficients were overlain on each symbol, with “red” symbols being used to denote a negative coefficient and “blue” symbols used to denote a positive coefficient.

## Results

### Physical Demands of Futsal

The analysis of absolute kinematic, mechanical, and metabolic variables revealed statistical differences between halves only for MP^TM^ with the first half requiring more energy expended by players than that in the second half (see Table 2).

**TABLE 2:** Descriptive statistics of absolute values observed during the first and second halves.

	Full match M ± SD	First half MD (IR)	Second half MD (IR)	Wilcoxon *W*	*p*
*Kinematics*					
Total distance covered	3,749 ± 1,123	1,875 (1,179)	1,674 (1,049)	1.37	0.18
Walking (0–6 km/h)	1,645.1 ± 442.9	792.7 (374.4)	759.4 (398.1)	0.72	0.48
Jogging (6–12 km/h)	1,321.5 ± 479.8	674.4 (465.4)	555.7 (547.9)	1.29	0.21
Running (12–18 km/h)	675.3 ± 298.1	328.6 (271.5)	317.5 (237.1)	1.60	0.12
Sprinting (>18 km/h)	134.9 ± 54.1	73.1 (56.8)	54.8 (55.7)	1.20	0.23
Maximum speed (km/h)	20.3 ± 1.7	20.4 (1.7)	20.6 (2.1)	0.33	0.74
*Mechanical*					
ACC (n/min)	87 ± 49	44 (43)	34 (36)	1.43	0.16
DEC (n/min)	80 ± 32	40 (35)	36 (33)	0.23	0.82
Jumps (n)	9 ± 4	3 (5)	4 (2)	0.33	0.75
Total impacts (n)	501 ± 388	219 (256)	194 (241)	1.33	0.19
Player load (a.u.)	72.1 ± 22.8	36.1 (19.1)	33.9 (14.7)	2.02	0.05
DSL (a.u.)	673.9 ± 247.7	314.9 (221.9)	340.6 (263.7)	−0.27	0.78
*Metabolic*					
Metabolic power (W/kg)	13.96 ± 3.09	7.9 (2.4)	6.5 (2.4)	3.73	0.00*
HMLD (W/kg)	655.79 ± 313.80	301.7 (252.9)	325.4 (263.7)	−0.95	0.35

*p < 0.001 significant difference; M, mean; SD, standard deviation; Md, median; and IR, interquartile range.

The analysis of relative kinematic, mechanical, and metabolic variables revealed differences between halves for running (12–18 km/h), with the second half revealing higher distance covered than the first half. Also, dynamic stress load (DSL) was higher in the second half than in the first half (see Table 3).

**TABLE 3:** Descriptive statistics of relative values observed during the first and second halves.

	Full match M ± SD	First half MD (IR)	Second half MD (IR)	Wilcoxon *W*	*p*
*Kinematics*					
Distance covered per minute	232 ± 71	216 (55)	229 (86)	−1.42	0.16
Walking per minute (0–6 km/h)	108.3 ± 51.5	92.5 (30.5)	110.8 (54.8)	−1.24	0.22
Jogging per minute (6–12 km/h)	76.5 ± 24.3	79.5 (16.5)	77.9 (17.9)	−0.54	0.59
Running per minute (12–18 km/h)	30.0 ± 19.2	15.7 (26.4)	38.6 (12.3)	−5.13	0.002*
Sprinting per minute (>18 km/h)	8.5 ± 7.9	7.4 (3.8)	7.3 (5.4)	−1.05	0.30
Sprints (n/min)	2 ± 1	2 (2)	2 (2)	0.84	0.41
*Mechanical*					
ACC (n/min)	5 ± 2	5.2 (2)	5.1 (2)	0.48	0.63
DEC (n/min)	5 ± 2	5 (2)	5 (2)	−0.77	0.44
Jumps (n/min)	0.8 ± 1.1	0.4 (0.5)	0.5 (0.9)	−1.76	0.09
Total impacts (n/min)	35 ± 35.2	29 (22.4)	30 (28.1)	0.00	1.00
Player load (a.u./min)	4.5 ± 2.3	4.1 (1.3)	4.3 (1.8)	−0.93	0.36
DSL (a.u./min)	15.0 ± 8.5	11.2 (13.4)	15.1 (13)	−2.73	0.004*
*Metabolic*					
Metabolic power per minute	6.9 ± 1.7	0.9 (0.6)	0.9 (0.8)	1.13	0.27
HMLD per minute	22.8 ± 10.6	22.2 (18.3)	23.7 (7.2)	−0.94	0.35

*p < 0.005 significant difference; M, mean; SD, standard deviation; Md, median; and IR, interquartile range.

### Clusters of Physical Profiles of Futsal Players

The cluster analysis classified the players into three distinct groups according to their physical profiles as higher, medium, and lower (Table 4), containing 4.5, 84.2, and 11.2% of the cases, respectively. The deceleration per minute (mechanical variable), walking per minute, sprinting per minute, jogging per minute, distance covered per minute, and MP^TM^ per minute were in descending order as variables that most contributed to the discrimination of the physical profiles of players. Deceleration per minute revealed significant differences between all profiles (*p* < 0.001), while the other reported variables only revealed significant differences between higher and medium and between higher and lower profiles (*p* < 0.05). High metabolic load distance (HMLD) was the most homogeneous variable, with a low predictor importance value.

**TABLE 4:** Classification of cluster physical profiles of futsal players.

Variables	Higher M ± SD	Medium M ± SD	Lower M ± SD	Sig. (*p*)	PI
*Kinematics*					
Distance covered per minute	364 ± 180	231 ± 46	185 ± 102	*^*,^^++^	0.992
Walking per minute (0–6 km/h)	249.2 ± 120.3	100 ± 29.5	114.7 ± 64.2	*^*,^^++^	1
Jogging per minute (6–12 km/h)	82.2 ± 67.3	80.5 ± 13.2	43.9 ± 37.8	^+, #^	0.997
Running per minute (12–18 km/h)	49.8 ± 53.5	30.8 ± 15.3	16.1 ± 17.6	+	0.825
Sprinting per minute (>18 km/h)	26.7 ± 31.5	8.2 ± 3.18	3.9 ± 3.3	*^*,^^++^	1
Sprints (n/min)	3.0 ± 1.0	2.0 ± 1.0	2.0 ± 1.0		0.126
*Mechanical*					
ACC (n/min)	5 ± 1	6 ± 2	3 ± 2	^##^	0.979
DEC (n/min)	10 ± 4	5 ± 1	2 ± 2	*^*,^^++,^^##^	1
No. of jumps (n/min)	1 ± 1.3	0.6 ± 0.6	0.5 ± 0.46		0.376
Total impacts (n/min)	42 ± 27	29 ± 16	75 ± 86	^##^	0.968
Player load (a.u./min)	4.3 ± 0.7	4.3 ± 1.3	6.2 ± 5.7		0.634
DSL (a.u./min)	20.7 ± 11	14.4 ± 7.9	17.2 ± 11.2		0.312
*Metabolic*					
Metabolic power per minute	16.9 ± 32.5	1.4 ± 2.6	1 ± 0.6	*^*,^^++^	0.989
HMLD per minute	24.8 ± 2.3	22.9 ± 11.2	21.3 ± 7.6		0.077

M, mean; SD, standard deviation; PI, predictor importance; *p < 0.05 higher with medium; **p < 0.001 higher with medium; **^+^**p < 0.05 higher with lower; **^++^**p < 0.001 higher with lower; **^#^**p < 0.05 medium with lower; and **^##^**p < 0.001 medium with lower.

### Collinearity Between EL Variables

**FIGURE 1:**
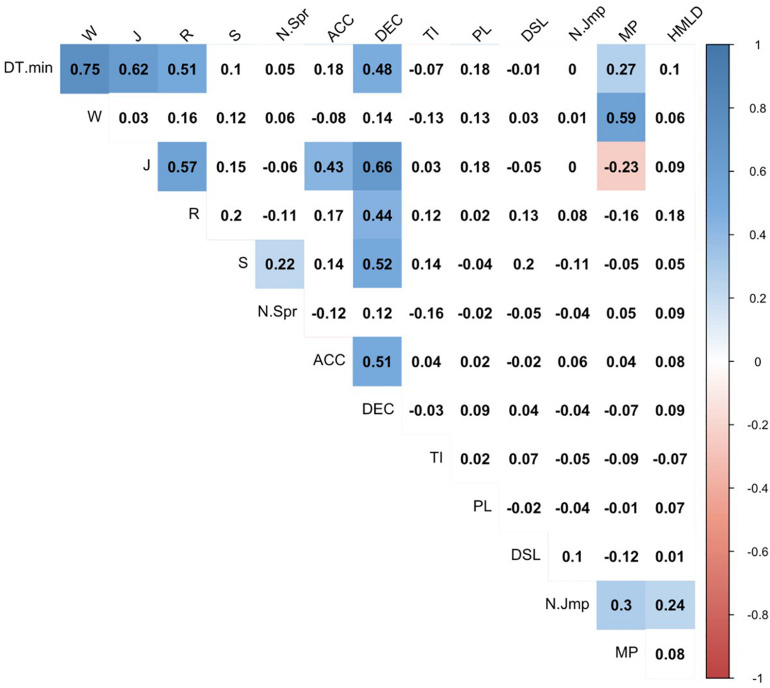
Correlation matrix between EL variables.

Figure 1 presents the level of magnitude of correlations between all the variables used in this study. The variables that showed the highest number of associations were distance covered per minute, deceleration per minute, MP per minute, and jogging per minute. In turn, total impacts per minute, PL^TM^ per min, DSL per minute, and number of jumps per minute did not show any type of correlation with others. The only negative correlation was found between MP^TM^ per min and jogging per minute.

## Discussion

The aim of the present study was to describe EL of futsal match play and identify the differences between the first and second halves. In addition, data were used to identify the external workload metrics that distinguish different futsal players’ profiles. At the end, the collinearity between external workload variables was also analyzed. To the best of our knowledge, this is the first study in official futsal competitions, and consequently, this is the first report on the kinematic, mechanic, and metabolic variables that characterize the physical load of futsal.

Generally, no meaningful differences were detected between halves. It was possible to identify three futsal players’ profile, based on the results of the following variables: deceleration per minute, walking per minute, sprinting per minute, jogging per minute, distance covered per minute, and MP^TM^ per minute. Also, the explorative analysis of the collinearity between EL variables allow us to identify the variables that have substantial impact to describe futsal physical demands in a simple way (Buchheit and Simpson, 2017). Distance covered per minute, deceleration per minute, jogging per minute, and MP^TM^ per minute were the variables that revealed a higher correlation with other variables.

### Futsal Game Characterization

In contrast to previous studies (Barbero-Alvarez et al., 2008; De Oliveira Bueno et al., 2014), no significant differences in EL were observed between the first and second halves of futsal matches. It seems to corroborate the most recent results in futsal in which no significant differences between the first and second halves were reported in IL indicators such as lactate and maximum heart rate values (Milioni et al., 2016). Such results relaunch the discussion about the capability of futsal players to maintain or even increase their physical performance during the entire match. The fact that futsal is characterized by unlimited substitutions and the score of the game may remain uncertain until near the end could be decisive for such results.

The comparison between absolute and relative workloads revealed different trends. Despite there being no statistical differences, there was a general decrease in the absolute values during the second half compared to the first half. In contrast, the relative values revealed a general increase in physical load per minute with clear higher values of running and DSL in the second half compared to the first half. These findings highlight the use of relative measures as more accurate information about the players’ intensity according to their participation in the game (Barbero-Alvarez et al., 2008; Whitehead et al., 2018). In this sense, it is clear that the ability to perform high-intensity actions remains during the entire match. In line with previous research, the average sprints (maximum speed) and the number of sprints remained stable between halves (Caetano et al., 2015). However, an interesting finding from this research was the higher distance covered per minute when compared to past research (Barbero-Alvarez et al., 2008; De Oliveira Bueno et al., 2014; Doğramaci et al., 2015). In fact, approximately twice more distance was covered per minute, and a higher number of sprints were performed.

The present study showed an average value of maximum speed of 20.3 km/h, with peak values of 22.6 km/h. The average values of peak sprinting speed is lower when compared with the values (23.8 km/h) reported in a previous report (de Pádua et al., 2017). Such results could be justified by a general increase in the work intensity of players in the last years, as well as by potential differences between leagues.

It is commonly accepted that mechanical variables such as accelerations and decelerations are the most important variables to be tracked in futsal, since they refer to a more neuromuscular- and biomechanical-oriented type of load (Buchheit, 2017). As in soccer, in futsal, due to the small space of action, the ability to accelerate and decelerate is considered decisive during critical actions, including changing direction, or rhythm in response to opponents’ actions, reaching the ball, and breaking movements to create space and generate or deny goal opportunities (Arruda et al., 2015). As far as we know, only one study reported mechanical and metabolic demands in futsal. However, it was developed with a female team from the Italian second division (Beato et al., 2017). Our results reported higher absolute values of accelerations and decelerations and similar values for metabolic demands in comparison with those of the female team (Beato et al., 2017).

### Futsal Player’s Profile

Futsal is characterized by a set of high-intensity efforts that require players with a high level of athletic performance in a multitude of physical abilities (Caetano et al., 2015; Miloski et al., 2016; Amani-Shalamzari et al., 2019). However, little information and consensus exist about the individual physical profile of futsal players. Identifying the variables that best discriminate the physical profiles of elite futsal players provides important data for the prescription and training periodization, thus highlighting the importance of analyzing and monitoring the physical demands of the match of each player according to their specific profile (Wilke et al., 2019; Rago et al., 2020).

Results of cluster analysis revealed three different groups with higher, medium, and lower levels of physical activity. Most of the players analyzed were classified as medium profile. The physical profiles of elite futsal players were discriminated by one mechanical variable (deceleration per minute), four kinematic variables (distance covered per minute, walking per minute, jogging per minute, and sprinting per minute), and lastly one metabolic variable (MP^TM^ per minute). Indeed, it seems that accelerations and decelerations could be used as reliable measures of different activity profiles of players (Cormack et al., 2014; Arruda et al., 2015). This method may allow grouping of players according to their physical and recovery profiles to understand if a slower or faster recovery can be related to different physical profiles (Wilke et al., 2019). Further research is required to improve the understanding between physical and technical–tactical profiles of play. In line with that, such information can also be used for the evaluation and development of young elite futsal players.

### External Workload Metrics: Collinearity Between Variables?

To improve the understanding of each variable and reduce the noise in the analysis, it is essential to simplify the results and improve their interpretation to provide reliable and useful information for coaches and strength-conditioning professionals (Buchheit and Simpson, 2017). For that, collinearity analysis between variables is crucial. Our results revealed that, in general, there were higher correlations between the distance covered per minute (kinematic), deceleration per minute (mechanical), and MP^TM^ per minute (metabolic), and other variables. In addition, the distance covered per minute and jogging per minute were the unique variables that revealed significant correlations with kinematic, mechanical, and metabolic variables. In the end, deceleration per minute revealed a high significant correlation with all kinematic variables except for walking per minute.

Regarding the analysis of kinematic variables, the distance covered per minute revealed significant correlations with walking per minute, jogging per minute, and running per minute, which means that distance covered per minute might be computed to generally represent all running speed thresholds between 0 and 18 km/h. So behind distance covered per minute, it is necessary to monitor distance covered above 18 km/h, in order to characterize all the speed thresholds considered. This evidence is in line with the importance and the need to individualize speed thresholds to provide an insight into players’ physical response to training and enable comparisons between player profiles (Rago et al., 2020). Analysis of mechanical variables revealed that deceleration per minute revealed a significant correlation with acceleration per minute. Thus, considering that deceleration per minute was highly associated with almost all kinematic variables, it may suggest that it is a more robust variable for analyzing the physical load of players during futsal training sessions and matches (Cormack et al., 2014). Therefore, it has a large association with the speed threshold of sprinting per minute, which is associated with an increase in heart rate variability, thus being able to play an important role as an indicator of good aerobic fitness (Buchheit, 2014).

The analysis of metabolic variables revealed that only MP^TM^ per minute demonstrated a positive correlation with kinematic variables (distance covered per minute and walking per minute) and a negative correlation with jogging per minute. This evidence suggests that MP^TM^ per minute might be less sensitive to peak demands. Thus, such a variable should be included in the analysis of physical demands of the futsal game as a complement to kinematic and mechanical variables that evaluate high match-play requirements (Polglaze and Hoppe, 2019). However, some caution while using this variable is advised as it does not agree with the literature (Gray et al., 2018).

## Limitations

As a possible limitation of the present investigation, we acknowledge that the sample size and number of matches should be larger in order to increase the power of the results (Lupo and Tessitore, 2016). In turn, the fact that it is a sample made up of elite players allows us to investigate the data of highly competitive demands. Thus, further research should be developed considering the influence of different contextual and situational variables in players’ EL, such as the evolution of match status and style of play (Lago-Peñas and Gómez-López, 2014). It would also be interesting to understand the worst-case scenarios (i.e., peak demands) for some EL variables, in order to prepare players for these specific moments of match play.

## Conclusion and Practical Applications

Overall, similar values were observed in most of the external variables between the first and second half. Interestingly, while the use of absolute results revealed a trend for a decrease from the first to the second half, in turn, the opposite was revealed when relative variables were analyzed according to the effective time of play of each player. Thus, relative measures to evaluate EL in futsal might be preferable, as it allow comparisons between studies and may also contribute to enhancing the comparison between players’ performance in both training sessions and matches.

The analysis of players’ profiles revealed that deceleration per minute, walking per minute, sprinting per minute, jogging per minute, distance covered per minute, and MP^TM^ per minute were the variables that best discriminated the profiles between players. Such results could help to better discriminate the individual training needs of each player and thus to adjust the prescription of training sessions. At the end, the explorative analysis of the collinearity between EL variables revealed that the distance covered per minute, deceleration per minute, and MP^TM^ per minute were the variables that revealed a higher correlation with other variables. Specifically, it was observed that distance covered per minute and deceleration per minute discriminate intensity while MP^TM^ per minute discriminated the volume of EL demands. Thus, to ensure a reliable analysis of EL demands in futsal, it is not necessary to measure all variables but rather consider those that better reflect the intensity of match play.

The transfer of this evidence to the training process is very significant; insofar as knowing the intensity of the match and which variables best characterize it, coaches can concretely manipulate and adjust the physical requirement of practice tasks during the microcycle to match demands in order to optimize players’ performance and reduce the risk of injury.

## Data Availability Statement

The raw data supporting the conclusions cannot be made available due to the restrictions defined by the participant clubs. Requests to access these datasets should be directed to joaonunorib@gmail.com.

## Ethics Statement

The studies involving human participants were reviewed and approved by local Ethics Committee of Universidade da Beira Interior (CE-UBI-Pj-2018-029). The patients/participants provided their written informed consent to participate in this study.

## Author Contributions

JR, JS, and BT contributed to the conception and design of the study. JR, BG, DC, and BT collected the data. BG performed the statistical analysis. JR and BT wrote the manuscript. JS, JB, DC, BG, and BT revised and finalized the manuscript. JR and BT organized the database. All authors contributed to the manuscript revision and read and approved the submitted version.

## Conflict of Interest

The authors declare that the research was conducted in the absence of any commercial or financial relationships that could be construed as a potential conflict of interest.
